# Enhanced bending strength and thermal conductivity in diamond/Al composites with B_4_C coating

**DOI:** 10.1038/s41598-018-29510-7

**Published:** 2018-07-23

**Authors:** Youhong Sun, Chi Zhang, Linkai He, Qingnan Meng, Bao-Chang Liu, Ke Gao, Jinhao Wu

**Affiliations:** 1 College of Construction Engineering, Jilin University, Changchun, 130026 People’s Republic of China; 2 Key Lab of Drilling and Exploitation Technology in Complex Conditions, Ministry of Land and Resources, Changchun, 130061 People’s Republic of China; 3 State Key Laboratory of Superhard Materials, Jilin University, Changchun, 130012 People’s Republic of China

## Abstract

Diamond/Al composites containing B_4_C-coated and uncoated diamond particles were prepared by powder metallurgy. The microstructure, bending strength and thermal conductivity were characterized considering the B_4_C addition and diamond fraction. The influence of B_4_C coating and fraction of diamond on both bending strength and thermal conductivity were investigated. The bending strength increased with decreasing diamond fraction. Moreover, addition of B_4_C coating led to an obvious increase in bending strength. The peak value at 261.2 MPa was achieved in the composite with 30 vt.% B_4_C-coated diamond particles, which was about twice of that for 30 vt.% uncoated diamond/Al composite (140.1 MPa). The thermal conductivity enhanced with the increase in diamond fraction, and the highest value (352.7 W/m·K) was obtained in the composite with 50 vt.% B_4_C-coated diamond particles. Plating B_4_C on diamond gave rise to the enhancement in bending strength and thermal conductivity for diamond/Al composites, because of the improvement of the interfacial bonding between diamond and aluminum matrix.

## Introduction

Metal matrix composites (MMCs) reinforced with diamond have achieved much attention in variety applications such as cut-off wheels and drills for concrete cutting, tunneling or oil exploration, due to its high hardness and grinding ability^1–5^. In addition, the excellent thermal conductivity (TC) and low thermal expansion of diamond reinforced MMCs make them attractive in the field of microelectronics and semiconductors^6–9^. The mechanical properties and thermal properties of composites are all determined by the interfacial bonding between diamond and metal matrix^10–16^. Recently, diamond reinforced aluminum or aluminum alloy composites have been proposed as candidate materials for above applications. However, the natural de-bonding between aluminum and the hexagonal diamond surfaces (with (1 1 1) orientations) is not conducive to obtain a strong interfacial bonding for the transfer of stress and heat^17^.

Coating strong carbide formers elements on diamond is an effective approach to optimize the interfacial bonding between diamond and metal matrix. The coatings form and bond with diamond during plating process, and alloy with metal matrix during sintering process. Zhang *et al*.^18^ studied the effects of diamond volume fraction and tungsten coating on the thermal properties of diamond/Al composites. The TC for the specimens with W-coated diamond particles exhibited above 90% of the theoretical values. Feng *et al*.^7^ reported that coating TiC was benefited to the enhancement of TC for diamond/Al composites, since Al_4_C_3_ and Ti_3_Al interfacial phase were formed during heating process, resulting in an improvement of combination between TiC-coated diamond and Al matrix. The Al_4_C_3_ is, however, a well-known brittle phase, which usually lead to a low strength for MMCs^19^. In addition, Wu *et al*. found^20^ that the forming of Ti_3_Al gave rise to a decrease in tensile strength for TiC-coated diamond reinforced Al. Thereby, for improving strength but not only TC, a more stable and strong interface was required for stress transfer.

Boron carbide (B_4_C) is widely used as a reinforcement particle in aluminum alloy for improving strength, hardness and wear resistance^21–25^. Zhang *et al*.^21^ investigated the effect of B_4_C content on mechanical properties of B_4_C/Al composite, and found that B_4_C particles contributed to the enhancement in hardness, bending strength and tensile strength. Rana *et al*.^25^ used 7075 Al as parent metal and B_4_C powder particles as reinforcement, and then fabricated 7075 Al/B_4_C surface composite through friction stir processing (FSP). A 100% improvement in wear resistance was achieved compared to the parent metal. These results indicated that Al/B_4_C exhibited a strong interface. Meanwhile, B_4_C bond with diamond via B-C covalent bond^26^. Therefore, pre-plating B_4_C coating on diamond was a potential effective method for increasing both strength and TC for diamond/Al composite.

In this work, we describe the great enhancement in bending strength and TC for aluminum composites reinforced with B_4_C-coated diamond particles. The diamond/Al composites with different volume fraction of B_4_C-coated and uncoated diamond particles were prepared by powder metallurgy. The B_4_C coating on diamond is conducive to obtain a dense diamond/Al composite due to the continual bonding interface. The enhancements of both mechanical and thermal properties are dependent on the porosity and the interfacial gap width between diamond and aluminum matrix.

## Results

**Figure 1:**
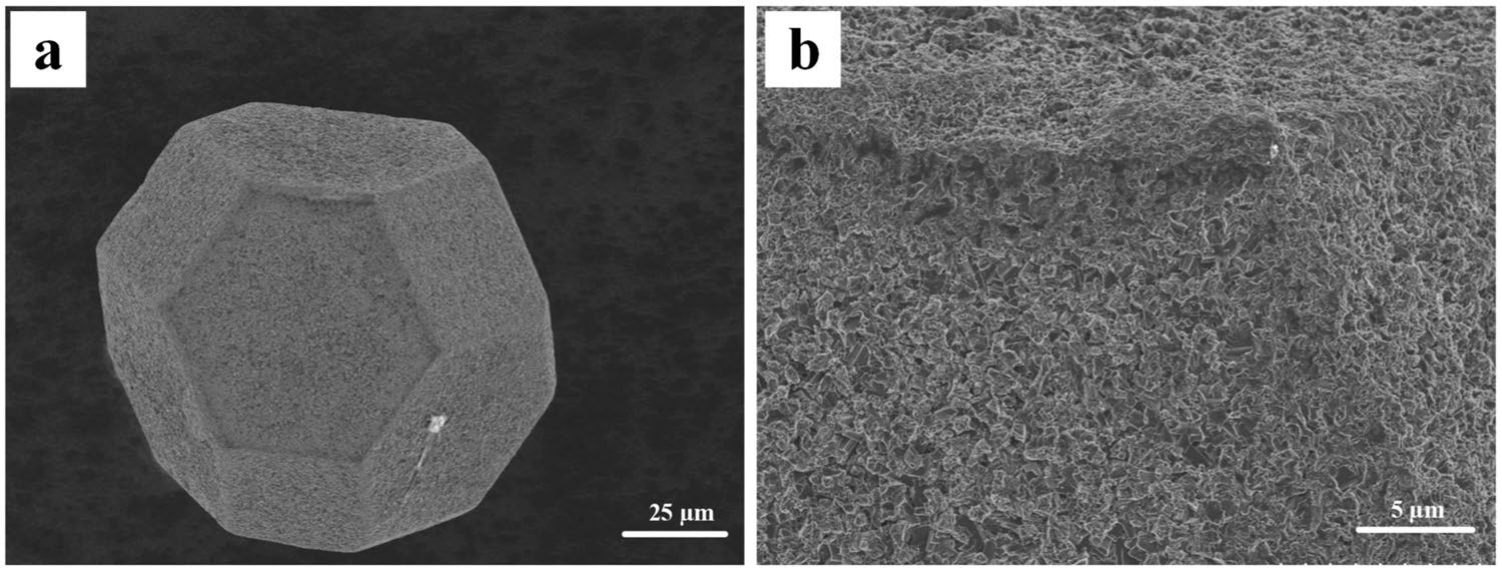
Morphologies for the B_4_C-coated diamond particles.

**Figure 2:**
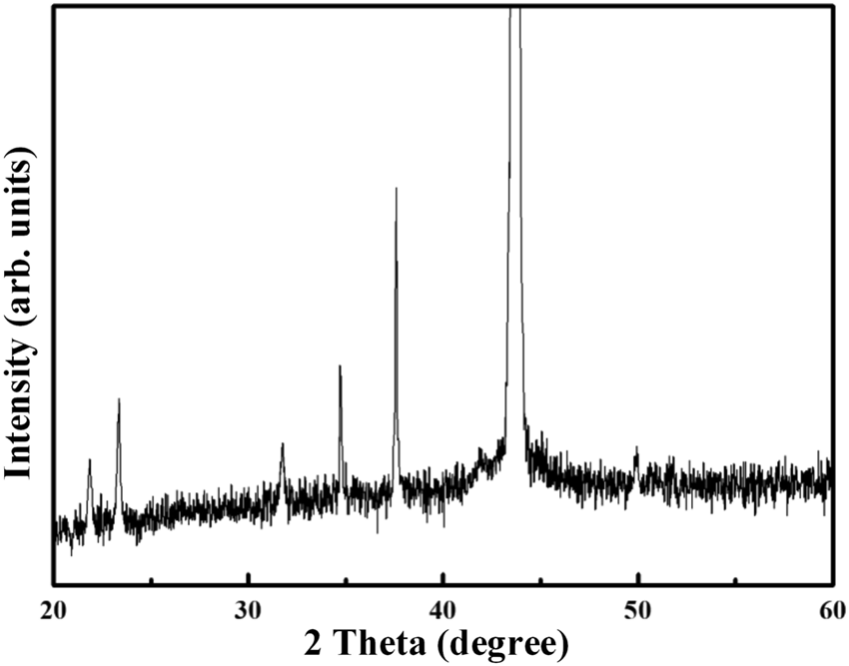
XRD spectrum for B_4_C-coated diamond particle.

The scanning electron microscopy (SEM) images for the B_4_C-coated diamond particles are shown in Fig. 1. For the coated diamond, a complete coverage of coating is achieved on all faces. The B_4_C layer is quite homogeneous, and the magnified image (Fig. 1b) indicates that the layer consists of submicron particles. The X-ray diffractogram (XRD) spectrum for the B_4_C-coated diamond particles is displayed in Fig. 2. As shown in Fig. 2, a high-intensity peak located at 43.9° is attributed to diamond (JCPDF#06–0675), which is partially truncated for clearly observing the other peaks for the coating on the diamond particle. Compared to the standard JCPDF#35–0798, the diffraction peaks located at 21.9°, 23.3°, 31.8°, 34.7°, 37.6° and 49.9° are ascribed to the typical B_4_C structure. The grain sizes of the B_4_C coating calculated from Williamson-Hall plot is 64 nm.

**Figure 3:**
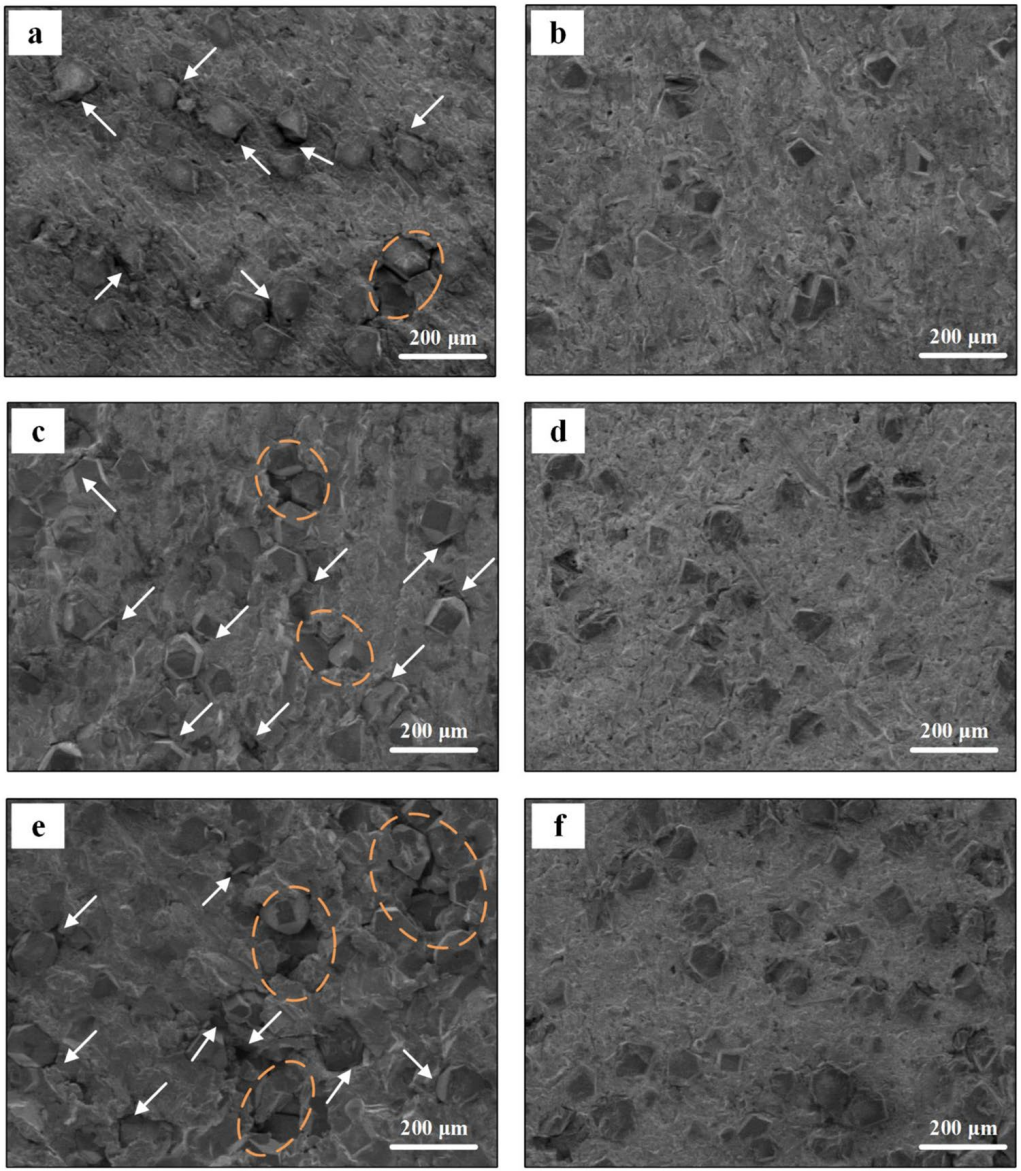
SEM micrographs for the diamond/Al composites with uncoated (**a**,**c**,**e**) and B_4_C-coated (**b**,**d**,**f**) diamond particles with fraction of 30 vt.% (**a**,**b**), 40 vt.% (**c**,**d**), and 50 vt.% (**e**,**f**), gap between diamond and aluminum matrix and un-wetting phenomenon are highlighted with white arrows and brown circles respectively.

SEM micrographs of the uncoated-diamond/Al (D1-3) and coated-diamond/Al (B1-3) composites are shown in Fig. 3. It can be seen that a quite homogeneous distribution of diamond particles is achieved and most corners of diamond particles remain. For the uncoated-diamond/Al composites (D1-3, Fig. 3a,c,e), wide gap between diamond and Al matrix is observed. Meanwhile, un-wetting phenomenon between diamond particles and Al matrix is existent on the surface of uncoated-diamond/Al composites. The wide gap and un-wetting phenomenon between diamond and Al are more significant with the increase in diamond fraction. In contrast, for the coated-diamond/Al composites (B1–3, Fig. 3b,d,f), both amount and width of gap between diamond particles and Al matrix exhibit an obvious decrease. Moreover, even for the composite with 50 vt.% B_4_C-coated diamond (B3, Fig. 3f), the un-wetting phenomenon is never observed. It indicates that the B_4_C coating gives rise to a good adhesion between diamond and Al matrix, which causes the densification of diamond/Al composites.

**Figure 4:**
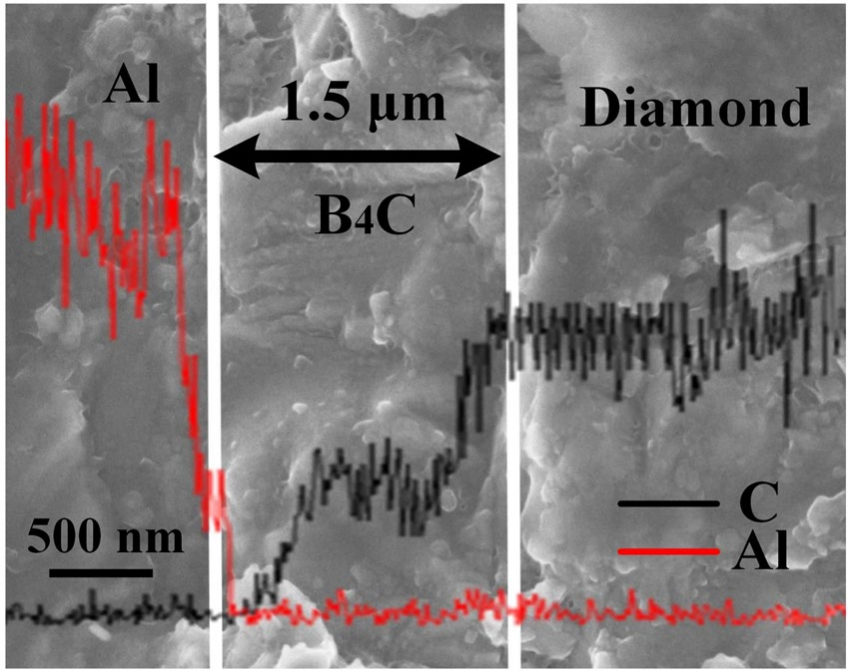
EDS interface line-scan in B_4_C-coated-diamond/Al composites.

The distribution of the composite elements (Al and C) in B_4_C-coated-diamond/Al composites is analyzed by EDS line-scanning and the results are shown in Fig. 4. Three distinct zones (Al zone, B_4_C layer and diamond zone) are presented in the figure. The thickness of the interfacial B_4_C layer is about 1.5 μm. When entering the interfacial B_4_C layer from the Al zone, a sharp decrease in intensity of the Al signal as well as an increase in intensity of C signal are observed. When entering the diamond zone from the interfacial B_4_C layer, a further increase in intensity of the C signal is observed, indicating the difference in the content of carbon between B_4_C and diamond.

**Figure 5:**
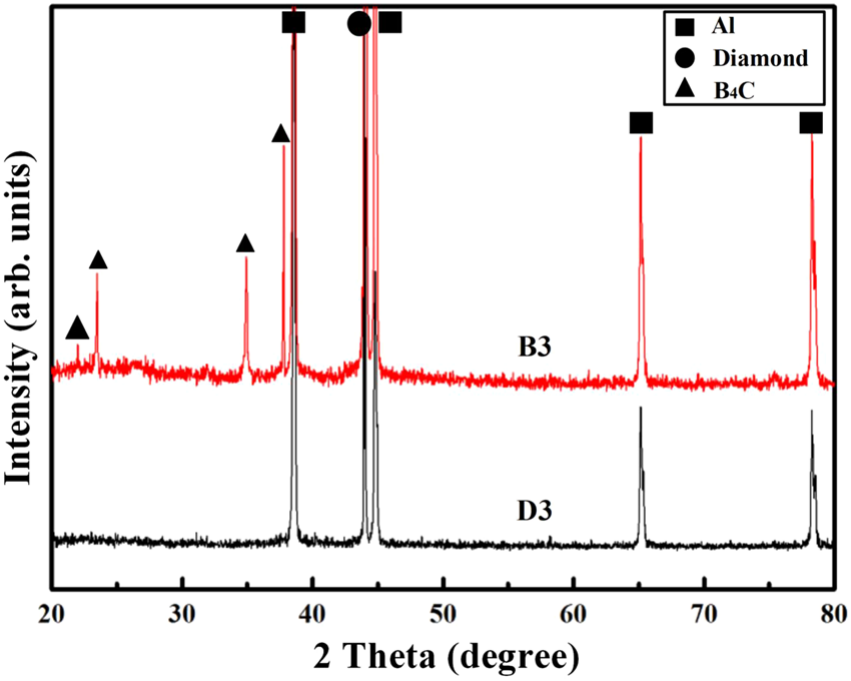
XRD patterns for diamond/Al composites containing 50 vt.% uncoated and B_4_C-coated diamond particles.

Figure 5 presents the XRD patterns for the diamond/Al composites with 50 vt.% uncoated and B_4_C-coated diamond particles (D3 and B3). Apart from the four typical aluminum peaks at 38.5°, 44.7°, 65.1° and 78.2° (JCPDF#65–2869), the diamond peak is observed at 43.9° (JCPDF#06–0675). For the 50 vt.% B_4_C-coated-diamond/Al composites (B3), typical B_4_C structure is confirmed by the peaks at 22.0°, 23.5°, 34.9° and 37.8° (JCPDF#35–0798).

**Figure 6:**
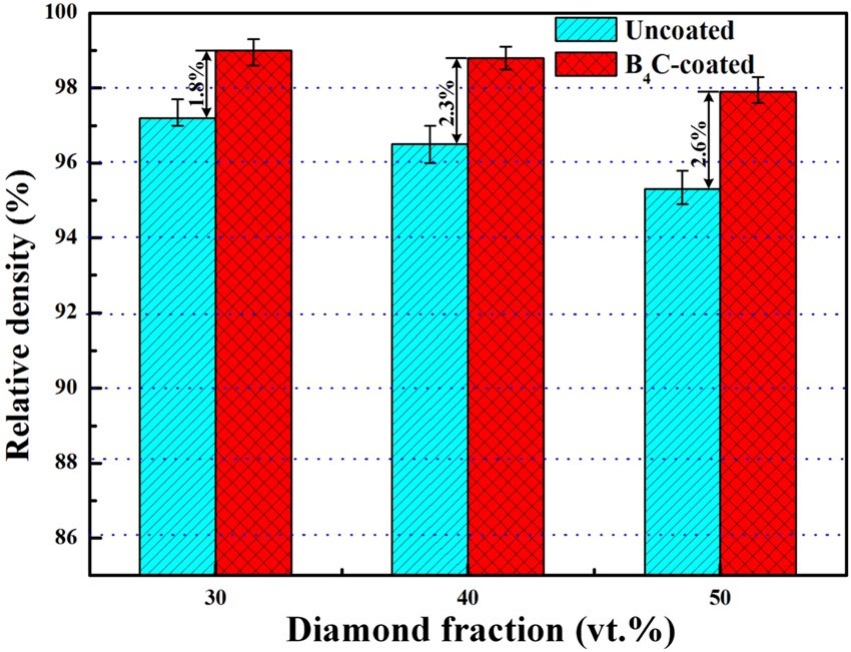
Relative density of the diamond/Al composites with uncoated and B_4_C-coated diamond particles as a function of fraction.

The theoretical density of the composte is determined by.

1 $${{\rm{\rho }}}_{theoretical}={{\rm{\rho }}}_{D}{V}_{D}+{{\rm{\rho }}}_{M}{V}_{M}$$

where *ρ*_*D*_ (3.52 g/cm^3 ^^27^) and *ρ*_*m*_ (2.70 g/cm^3 ^^27^) is theoretical density of diamond and aluminum matrix, respectively. *V*_*D*_ and *V*_*M*_ are the volume fraction of diamond particles and aluminum matrix respectively. Thereby, the theoretical density for composite with 30, 40 and 50 vt.% diamond is 2.95, 3.03 and 3.11 g/cm^3^, respectively. With increasing diamond from 30 to 50 vt.%, the density of uncoated-diamond/Al composites is 2.86, 2.92 and 2.96 g/cm^3^ (D1-3), which is quite lower than the corresponding theoretical density. In contrast, plating B_4_C coating on diamond gives rise to dense composites. The composite with 30 vt.% B_4_C-coated diamond (B1) exhibits a density of 2.92 g/cm^3^, which is quite close to the theoretical density. The composite with 50 vt.% B_4_C-coated diamond particles (B3) still maintains a high density (3.05 g/cm^3^), which is denser than all uncoated-diamond/Al composites (D1-3). The relative density ${{\rm{\rho }}}_{relative}$ is determined by.

2 $${{\rm{\rho }}}_{relative}={{\rm{\rho }}}_{measured}/{{\rm{\rho }}}_{theoretical}$$

where ${{\rm{\rho }}}_{measured}$ is the measured density. The relative densities for Al/diamond composites with different diamond fractions of uncoated and B_4_C-coated diamond particles are shown in Fig. 6. As shown in Fig. 6, the relative densities for composites with both uncoated and B_4_C coated diamond particles decrease with the increase in diamond fraction, whereas the relative densities for composites with B_4_C coated diamond particles (>97.9%) are higher than those for composites with uncoated diamonds (up to 97.2%). Furthermore, by comparing relative density for the B_4_C-coated-diamond/Al with that for the corresponding uncoated-diamond/Al composite, the improvement in relative density increases from 1.8 to 2.6% with increasing diamonds fraction from 30 to 50 vt.%, meaning that plating B_4_C coating on diamond is benefit to the densification of diamond/Al composite.

**Figure 7:**
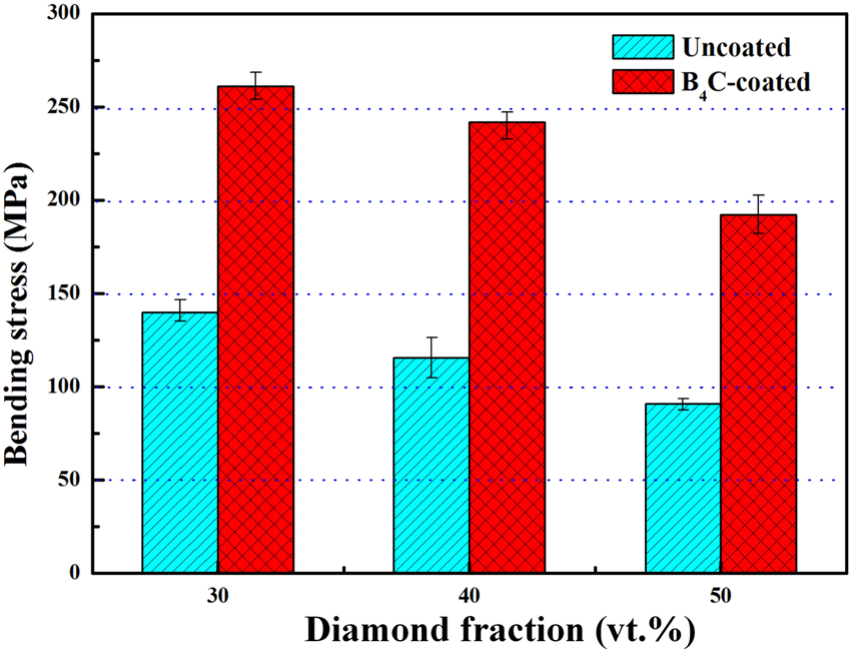
The bending strength of B_4_C-coated and uncoated diamond/Al composites with different diamond fractions obtained by three-point bending tests.

Figure 7 displays the bending strength obtained by the three-point bending tests. For all composites, the increase in diamond fraction results in a decrease in bending strength. The bending strength for uncoated-diamond/Al composite decreases from 140.1 to 90.8 MPa with increasing diamond fraction from 30 to 50 vt.%. It is worth noted that the bending strength for the B_4_C-coated diamond/Al composites are much higher than that for the uncoated diamond/Al composites. The composite with 30 vt. % B_4_C coated diamond particles (B1) exhibits the largest bending strength (261.2 MPa), which increases by 86.4% compared with the corresponding composite with 30 vt. % uncoated diamond particles (D1). In addition, the bending strength for composite with 50 vt. % B_4_C coated diamond particles (B3) is 192.4 MPa, which is over twice of that for composite with 50 vt. % uncoated diamond particles (D3, 90.8 MPa) and even larger than that for composite with 30 vt. % uncoated diamond particles (D1, 140.1 MPa).

**Figure 8:**
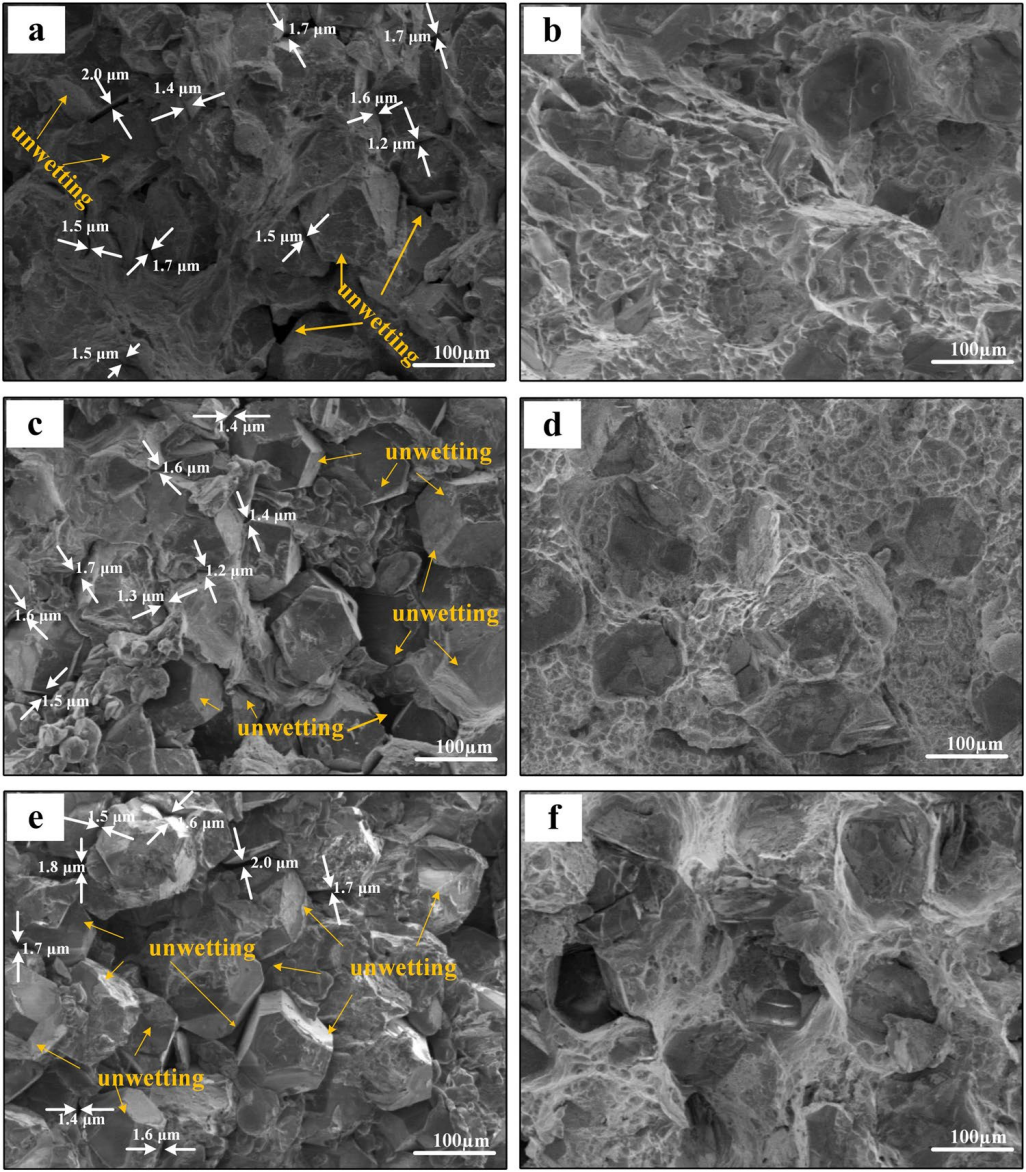
SEM images for the bending fracture surfaces of the diamond/Al composites with uncoated (**a**,**c**,**e**) and B_4_C-coated (**b**,**d**,**f**) diamond particles with fractions of 30 vt.% (**a**,**b**), 40 vt.% (**c**,**d**), and 50 vt.% (**e**,**f**).

**Figure 9:**
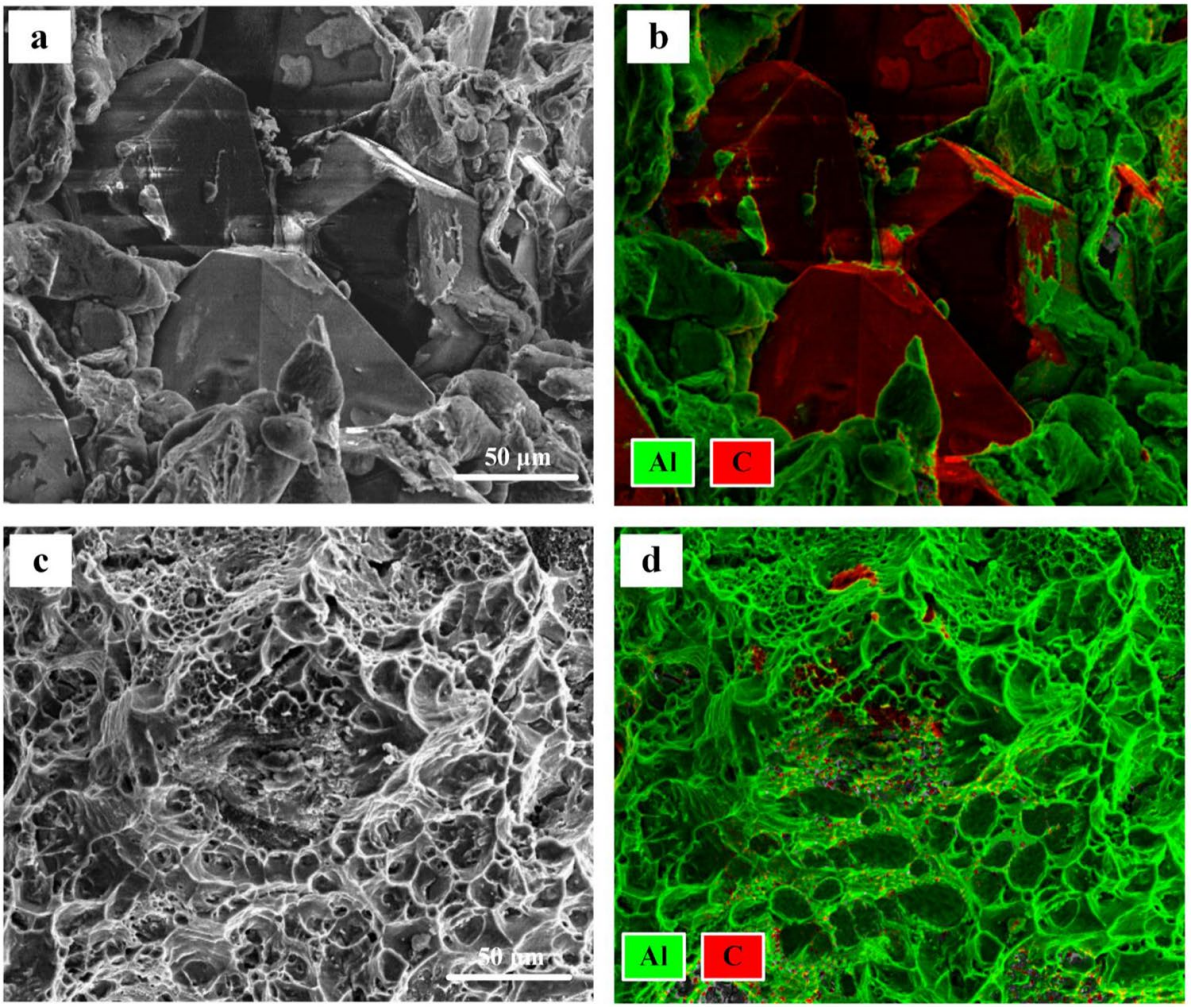
The elements distribution maps of the samples D3 and B3.

SEM micrographs for bending fracture surfaces of the diamond/Al composites with uncoated (D1-3) and B_4_C-coated (B1-3) diamond particles are given in Fig. 8. For the uncoated-diamond/Al composites (D1-3, Fig. 8a,c,e), large amounts of wide gap around diamond particles are observed, which is marked by white arrows. Meanwhile, un-wetting phenomenon between aluminum matrix and diamond particles is existent, which is highlighted by brown arrows. For the B_4_C-coated-diamond/Al composites (B1-3, Fig. 8b,d,f), no obvious gap around diamond particles is found. EDS mapping was used to evaluate the bonding condition between diamond particles and aluminum matrix. Figure 9 presents the elements distribution maps of diamond/Al composites containing 50 vt.% uncoated and B_4_C-coated diamond particles. As shown in Fig. 9a,b, obvious gap at the interface boundary is observed for the uncoated-diamond/Al composite (D3). Meanwhile, most surfaces of the diamond particle remain naked without aluminum matrix surrounded, indicating a poor interfacial bonding caused by the un-wetting between diamond and aluminum matrix. It implies the fracture of composite D3 occurred from interface between diamond and Al matrix, because of the poor interfacial bonding. According to the elements distribution map of sample B3 (Fig. 9c,d), the adhesion of the aluminum matrix to the diamond particle is remarkably improved. Most diamond surfaces are found to be embedded in the aluminum matrix, while the exposed part is covered by large amounts of aluminum dimples. It indicates that ductile fracture of Al matrix has replaced interfacial fracture to be the dominant fracture mode in composite B3, since better wettability and interfacial bonding between diamond particles and aluminum achieved by plating B_4_C.

**Figure 10:**
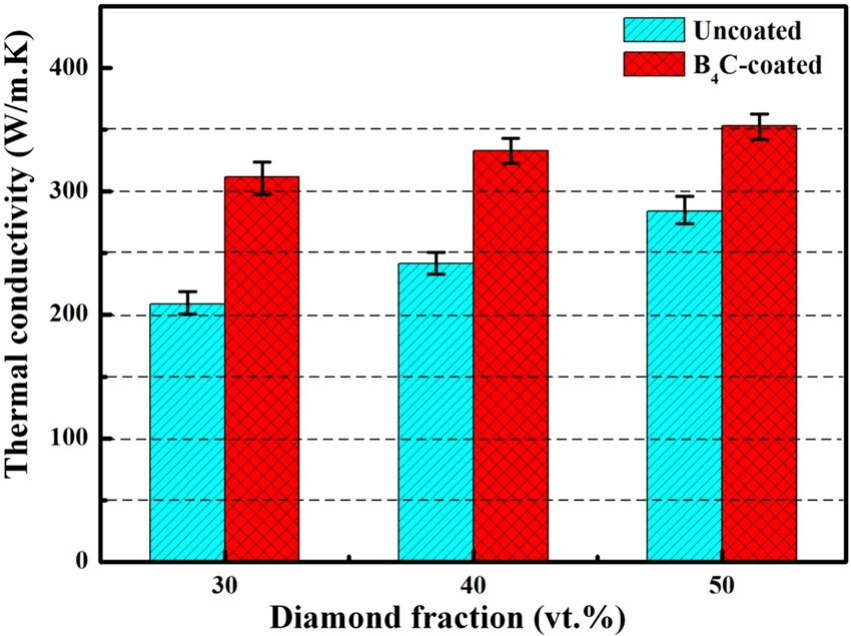
Thermal conductivity of diamond/Al composites with uncoated and B_4_C-coated diamond particles.

Figure 10 shows the TC of diamond/Al composites with uncoated (D1-3) and B_4_C-coated (B1-3) diamond particles. As shown in Fig. 10, uncoated-diamond/Al composites (D1-3) exhibit lower TC, and the TC for sample D1 (208.4 W/m·K) is even lower than that for pure aluminum (237 W/m·K^28^). With increasing diamond fraction from 30 to 50 vt.%, the TC for uncoated-diamond/Al composite increases from 208.4 to 283.8 W/m·K, meanwhile that for B_4_C-coated-diamond/Al composite increases from 311.4 to 352.7 W/m·K. Moreover, it should be noted that B_4_C coating gives rise to an obvious increase in thermal conductivity, because the TC for B_4_C-coated diamond/Al composites are much higher than those for uncoated diamond/Al composites. The TC for composite containing 30 vt.% B_4_C-coated diamond particles (B1) even performs better than that for the composite with 50 vt.% uncoated diamond particles (D1).

## Discussion

**Figure 11:**
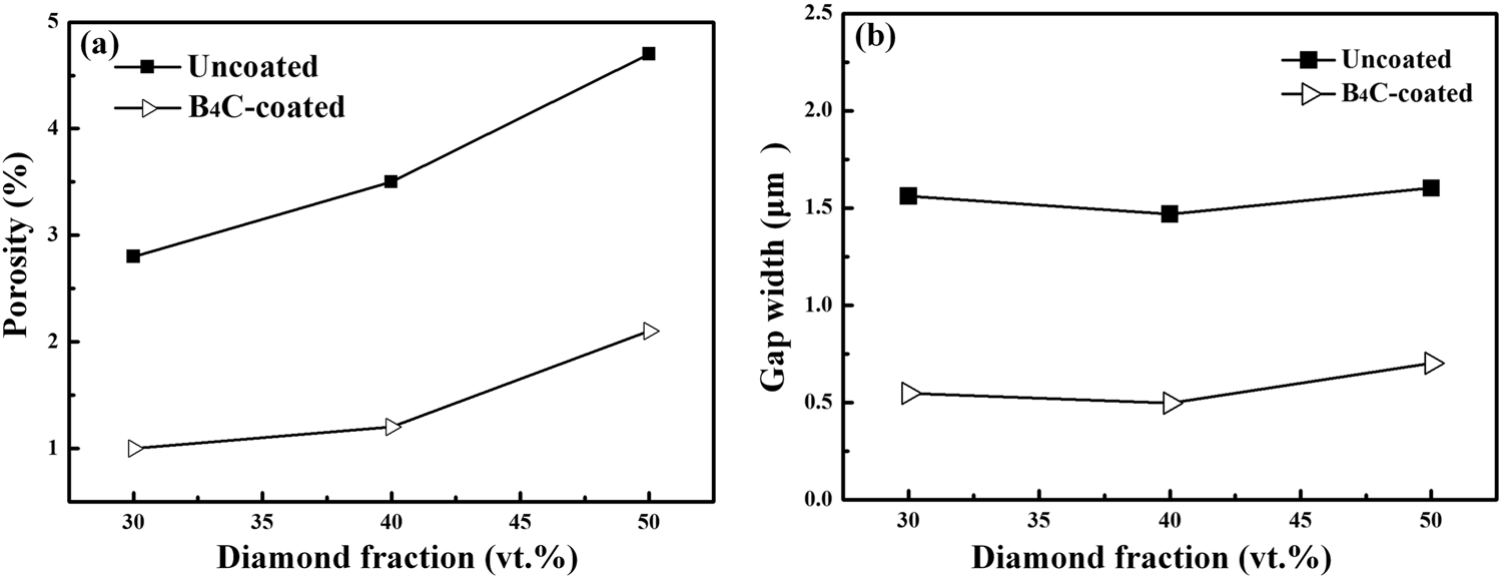
(**a**) Porosity and (**b**) calculated gap width for uncoated and B_4_C-coated diamond/Al composites with different diamond fractions.

As shown in Fig. 6, the relative density for uncoated diamond/Al composites (D1-3) is lower than that for B_4_C-coated diamond/Al composites (B1-3) at each diamond fraction, indicating that a larger number of pores exist in samples D1-3. Meanwhile, SEM images for bending fracture surface show that wider interfacial gap is observed between uncoated diamond and aluminum matrix. The separation between diamond and aluminum is caused during the cooling process by the large difference in expansion coefficients between aluminum (23.0 × 10^−6^ K^−1 ^^29^) and carbon materials (1.0 × 10^−6^ K^−1 ^^27^). Therefore, the low density for uncoated diamond/Al composites is attributed to large amounts of wide gap around diamond particles. Moreover, the decrease in relative density for uncoated diamond/Al composite with the increase in diamond fraction is caused by the larger amount of wide gap provided by longer interface. Assuming that the diamond used in this study is isotropic spherical particle, the average gap width (x) can be evaluated by

3 $$\frac{{a}^{3}}{{(a+x)}^{3}}=\frac{(1-{V}_{p}){V}_{D}}{(1-{V}_{p}){V}_{D}+{V}_{p}}$$

4 $${V}_{P}=1-{\rho }_{relative}$$

where a is the radius of diamond particle, *V*_*D*_ is the volume fraction of diamond particle, and *V*_*P*_ is porosity (Fig. 11a). As displayed in Fig. 11b, plating B_4_C coating on diamond reinforcement gives rise to a decrease in average gap width from 1.54 to 0.58 μm, which agrees well with SEM results (Fig. 8). B_4_C interlayer is benefited to relieve the interfacial thermal stress between aluminum and diamond during the cooling process due to the modest expansion coefficients of B_4_C (5.65 × 10^−6^ K^−1 ^^30^). In addition, the improvement rate attributed to B_4_C interface is more significant for the composite with larger diamond fraction. Therefore, we suggest that the formation of B_4_C is benefited to the densification of diamond/Al composite.

**Figure 12:**
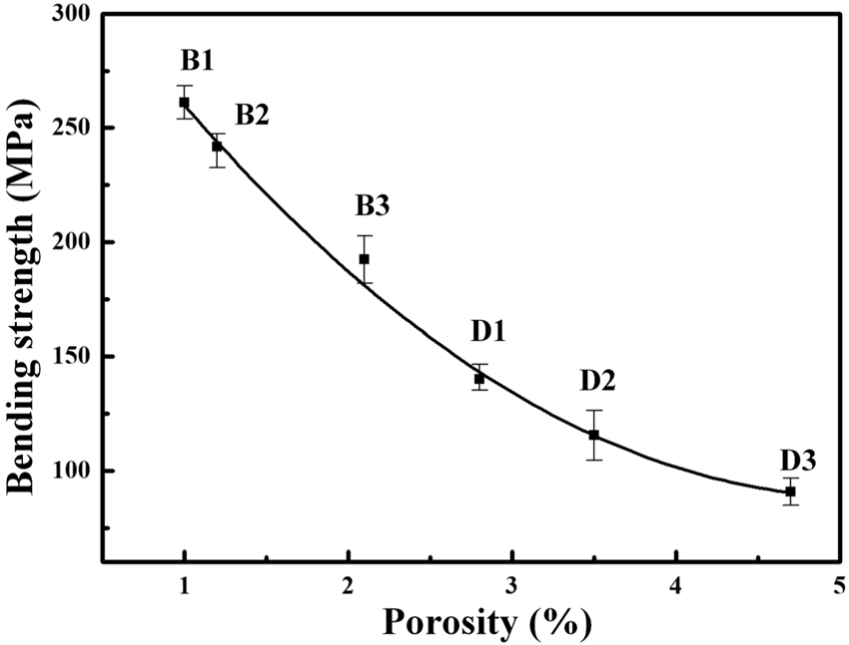
The relationship between bending and porosity.

Figure 12 summarizes the relationship between bending strength and porosity. It can be seen that the bending strength is strongly dependent on the porosity of diamond/Al composites. Together with the average gap width (Fig. 11) and SEM images (Fig. 8), uncoated-diamond/Al composites (D1-3) are fabricated with high porosity along with a large number of gap and pores, which contributes to the extension of the crack under stress. Therefore, the significant decrease in bending strength for uncoated diamond/Al composites with the increase in diamond fraction (as shown in Fig. 7) is due to that the larger diamond fraction results in a longer weak interface and larger number of gap and pores. For the B_4_C-coated diamond/Al composites (B1-3), plating B_4_C on diamond contributes to improve the wettability between diamond and aluminum matrix and optimize the interface structure. B_4_C coating is benefited to the increase in bending strength due to the decrease in average gap width and porosity. For the 30 vt.% B_4_C-coated-diamond/Al composite (B1), no obvious gap between diamond and aluminum matrix is observed and the highest bending strength is achieved, because the continual interface is conducive to distribution of stress. Thereby the enhancement is more evident for the composite with larger diamond fraction, since longer weak interface between naked diamond and Al matrix is enhanced by B_4_C interface. Furthermore, the 50 vt.% B_4_C-coated-diamond/Al composite (B3) still maintain a high bending strength which is even larger than the bending strength for the composite with 30 vt.% uncoated diamond particles (D1), because the number of gap and pores for B3 is lower than that for D1 via adding a B_4_C interlayer between diamond and Al matrix.

Furthermore, the EDS mapping results reveal the conversion of fracture behavior of diamond/Al composites after plating B_4_C on diamond particles. The dominant position of interfacial fracture (Fig. 9b) suggests a weak interfacial bonding between diamond and Al matrix for uncoated-diamond/Al composites, which is not benefited to the stress transfer and leads to a lower bending strength and brittle fracture. In contrast, for the composite with B_4_C-coated diamond (e.g. B3), large number of dimples are observed on the surface of diamond, which is confirmed to be Al by EDS analysis. It demonstrates that the fracture mode of B_4_C-coated-diamond/Al composite is converted to ductile fracture, and a strong interfacial bonding between diamond particles and aluminum are achieved by plating B_4_C.

As shown in Fig. 10, plating B_4_C on diamond particles contributes to a significant increase in TC for diamond/Al composites. The TC for composites depends on many factors, such as component, reinforcement fraction and size, distribution, and interfacial bonding between the matrix and reinforcement^18^. To better understand the thermal conductivity behavior of diamond/Al composites, it is necessary to compare experimental results with theoretical predictions. Among those models developed by researchers, the Hasselman-Johnson (H-J) model^31^ was used to estimate effective thermal conductivity of composites ${K}_{c}$ by taking interfacial thermal barrier into consideration.

5 $${K}_{C}={K}_{m}(\frac{2(\frac{{K}_{r}}{{K}_{m}}-\frac{{K}_{r}}{a{h}_{c}}-1){V}_{r}+\frac{{K}_{r}}{{K}_{m}}+\frac{2{K}_{r}}{a{h}_{c}}+2}{(1-\frac{{K}_{r}}{{K}_{m}}+\frac{{K}_{r}}{a{h}_{c}}){V}_{r}+\frac{{K}_{r}}{{K}_{m}}+\frac{2{K}_{r}}{a{h}_{c}}+2})$$

where K_m_ and K_r_ are TC of matrix and reinforcement particles respectively (K_Al_ = 237 W/m·K^32^, K_diamond_ = 1350 W/m·K^7^), a is the radius of reinforcement particle. The interfacial thermal conductance h_c_ is identified as

6 $${h}_{C}=\frac{1}{2}{\rho }_{m}{c}_{m}\frac{{{v}_{m}}^{3}}{{{v}_{r}}^{2}}\frac{{\rho }_{m}{\rho }_{r}{v}_{m}{v}_{r}}{{({\rho }_{m}{v}_{m}+{\rho }_{r}{v}_{r})}^{2}}$$

where *ρ*_*m*_ and *ρ*_*r*_ are theoretical density of matrix and reinforcement particle respectively, *v*_*m*_ and *v*_*r*_ are phonon velocity in matrix and reinforcement particle respectively (*v*_*Al*_ = 3040 m/s, ${v}_{diamond}$= 13924 m/s^26^), and *c*_*m*_ is the specific heat of matrix (*C*_*Al*_ = 880 J/kg·K^7^).

**Figure 13:**
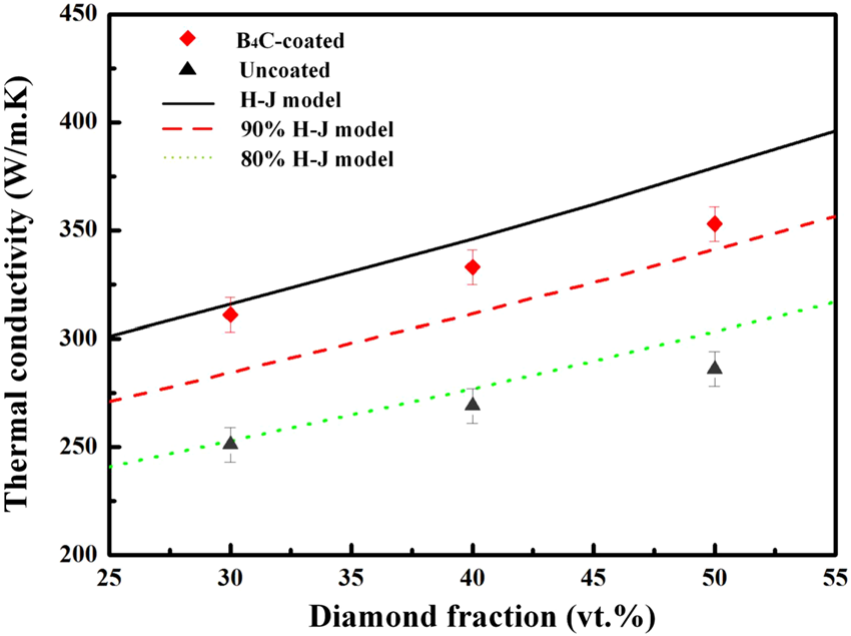
Variation in thermal conductivity of the diamond/Al composites containing uncoated and B_4_C-coated diamond particles.

The experimental data and theoretical prediction of the TC of diamond/Al composites with uncoated and B_4_C-coated diamond particles are displayed in Fig. 13. As shown in Fig. 13, measured TCs of B_4_C-coated-diamond/Al composites are close to theoretical values, whereas for uncoated-diamond/Al composites measured TCs exhibit a clear difference with theoretical results. Near 100% of the theoretical value (316.1 W/m·K) is reached for the 30 vt.% B_4_C-coated-diamond/Al composite (B1, 311.4 W/m·K), while only around 80% of the theoretical value is reached for D1 with uncoated diamond particles. In addition, it is worth noted in Fig. 13 that with the diamond fraction increasing, the deviation between the measured TC and theoretical value becomes larger, which is hard to be explained through the H-J model.

**Figure 14:**
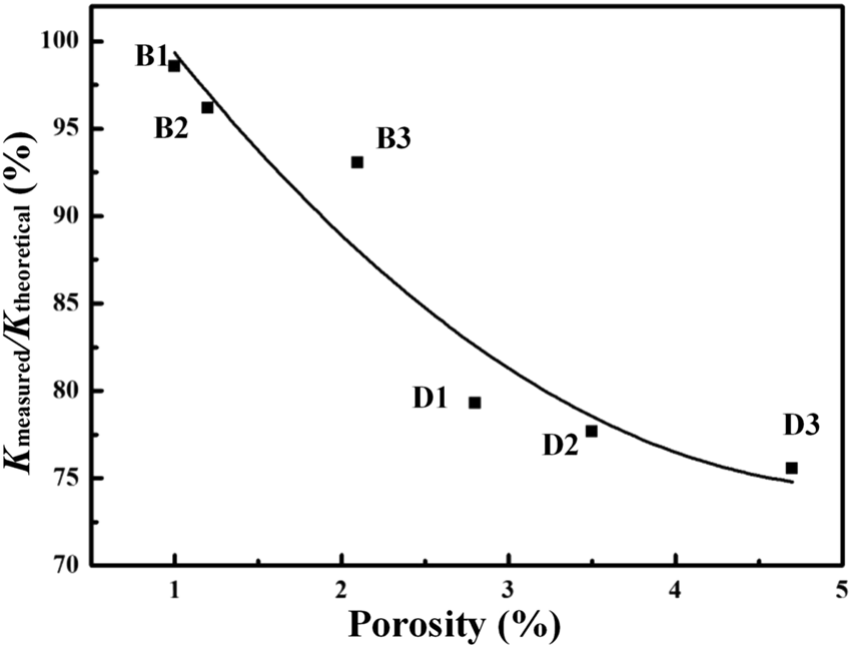
The relationship between K_measured_/K_theoretical_ and porosity.

Although many factors are taken in the H-J model to evaluate the TC of composites, the porosity in theses specimens is not taken into consideration. It is well known that air (or vacuum) is an excellent heat insulation layer. Furthermore, the influence of porosity on TC was investigated in diamond/Cu composites^26^. Therefore, the existence of gap in composite contributed to the deterioration in TC of composite, leading to a larger deviation between measured and theoretical results. To further understand the effect of porosity on the TC of diamond/Al composites, The relationship between K_measured_/K_theoretical_ and porosity for all composites is displayed in Fig. 14. It reveals that K_measured_/K_theoretical_ is highly dependent on the porosity of diamond/Al composites. The measured TC of diamond/Al composite is closer to the theoretical prediction by the H-J model with a higher density. The relative density of diamond/Al composites (Fig. 6) suggests that the relative density decreases with increasing diamond fraction. Thereby, lower fraction of diamond in composite gives rise to an increase in K_measured_/K_theoretical_ (Fig. 14) because of the decrease in porosity. In addition, combined with densification and TC analyze, the addition of B_4_C interlayer is contributed to the densification of composite, and benefit to achieve a continual interface between diamond reinforcement and Al matrix, which gives rise to an obvious enhancement in thermal conductivity. Therefore, the composites with a B_4_C interlayer display higher TCs (Fig. 10) and larger K_measured_/K_theoretical_ (Fig. 14).

To summarize, diamond/Al composites with different fraction of uncoated and B_4_C-coated diamond particles were prepared by powder metallurgy. Interfacial bonding and porosity were found to be the key factors in determining the properties of diamond/Al composites. The addition of B_4_C coating was benefit to the decrease in interfacial gap between diamond and Al, which gave rise to a dense composite. In addition, both bending strength and thermal conductivity of composites were dependent on the interfacial gap width between diamond and Al matrix. The bending strength for composites with B_4_C coated diamond was about twice of that for composites with un-coated diamond. Meanwhile, the specimens with B_4_C-coated diamond particles exhibited high TC (up to 352.7 W/m·K), even the sample with 30 vt.% B_4_C-coated diamond exhibited a TC as 311.4 W/m·K, which is quite larger than that for composite with un-coated diamond (up to 283.8 W/m·K) and pure Al (237 W/m·K^28^).

## Method

As the composite matrix, the pure aluminum powder with an average size of 74 μm was provided from Shanghai Chaowei Nanotechnology Co. Ltd., China. Synthetic HPHT diamond particles (HSD90, particle size 140/170 mesh (89~104 μm), Henan huanghe whirlwind international Co., Ltd., China) with cubic-octahedral monocrystalline were used as the reinforcement. For forming boron carbide (B_4_C) coating on diamond particles, the mixture of boron (B), boric acid (H_3_BO_3_) and diamond particles was heated in a tube furnace at 1200 $^\circ {\rm{C}}$ for 6 h in Ar atmosphere. The method was described in detail in the previous work^26^. Different fraction of diamonds (30, 40 and 50 vt.%) were mechanically mixed with aluminum powder at room temperature. For comparison, the uncoated and B_4_C-coated diamond particles were both applied. The powder mixtures were then subjected to vacuum hot pressing in a graphite die. The furnace was heated up to 600 °C at a heating speed of 10 °C/min, then held for 30 min under a uniaxial pressure at 30 MPa in order to ensure the density. The temperature during hot pressing was monitored through a thermocouple inserted into the graphite die. After sintering, the specimens were cooled in furnace to the room temperature. The vacuum was needed before the specimens being cooled to room temperature. The composites with different fractions of B_4_C-coated and un-coated diamond reinforcements were referred as B1-3 and D1-3 respectively, as shown in Table 1.

**Table 1:** Sample numbers for B_4_C-coated and uncoated-diamond/Al composites.

B_4_C-coated		Uncoated	
Volume fraction (%)	Sample number	Volume fraction (%)	Sample number
30	B1	30	D1
40	B2	40	D2
50	B3	50	D3

To investigated the microstructure of coating and the interfacial product, X-ray diffraction (XRD) was performed on a Bruker D8 with a Cu K ${\rm{\alpha }}$ source in the step mode from 20° to 80° at a scanning speed of 0.08 °/s. A Hitachi S-4800 scanning electron microscope (SEM) was used to characterize the distribution of diamond particles in composites. The density of composites was measured by a high precision ceramic porosity volume density tester (Dahometer, DE-120M) using Archimedes method^29^. Three-point bending strength was measured on the specimens with a dimension of 5 × 8 × 50 mm. The bending test was carried out with an initial speed of $0.50$ mm/min using an electronic universal test machine (DDL 100, CIMACH, Changchun, China). The morphologies of fracture surfaces were also obtained by SEM. X-ray energy dispersive spectrometer (EDS) attached to the SEM equipment was applied to analyze the elements distribution on the fracture surface of composites. Thermal diffusivity of the composites at room temperature was measured by a laser flash method by a NETZSCH LFA427/3/G thermal physical testing instrument. Specific heat of the composites was derived from the theoretical value calculated according to the rule of mixture (ROM). Finally, the thermal conductivity was calculated by the product of density, thermal diffusivity and specific heat according to the following equation^28^:

7 $${\rm{\lambda }}={\rm{\alpha }}{\rho }_{measured}C$$

where α was thermal diffusivity, ${\rho }_{measured}$ was the measured density of composites and C was specific heat. Detailed results were shown in Table 2.

**Table 2:** Thermal conductivity measurements of diamond/Al composites.

Sample	Density (g/cm^3^)	Specific heat (J/g·K)	Thermal diffusivity (mm^2^/s)	Thermal conductivity (W/m·K)
D1	2.86	0.76	95.9	208.4
D2	2.92	0.71	115.9	240.3
D3	2.96	0.67	144.4	286.4
B1	2.92	0.77	139.0	312.5
B2	2.99	0.73	152.3	332.4
B3	3.04	0.69	168.6	353.7

## References

[1] Li, M. *et al*. Fabrication of Fe-Based Diamond Composites by Pressureless Infiltration. *Materials* **9**, 10.3390/ma9121006 (2016). 10.3390/ma9121006 PMC5457001 28774124

[2] Sun, Y. *et al*. The Effect of ZrO_2_ Nanoparticles on the Microstructure and Properties of Sintered WC-Bronze-Based Diamond Composites. *Materials* **9**, 10.3390/ma9050343 (2016). 10.3390/ma9050343 PMC5503043 28773469

[3] Webb SW (1999). Diamond retention in sintered cobalt bonds for stone cutting and drilling. Diamond and Related Materials.

[4] Weidenmann KA, Tavangar R, Weber L (2009). Mechanical behaviour of diamond reinforced metals. Materials Science and Engineering: A.

[5] Xu X, Tie X, Wu H (2007). The effects of a Ti coating on the performance of metal-bonded diamond composites containing rare earth. International Journal of Refractory Metals and Hard Materials.

[6] Ekimov EA, Suetin NV, Popovich AF, Ralchenko VG (2008). Thermal conductivity of diamond composites sintered under high pressures. Diamond and Related Materials.

[7] Feng H, Yu JK, Tan W (2010). Microstructure and thermal properties of diamond/aluminum composites with TiC coating on diamond particles. Materials Chemistry and Physics.

[8] Kidalov SV, Shakhov FM, Vul AY (2008). Thermal conductivity of sintered nanodiamonds and microdiamonds. Diamond and Related Materials.

[9] Xue C, Yu JK, Zhu XM (2011). Thermal properties of diamond/SiC/Al composites with high volume fractions. Materials & Design.

[10] Chung C-Y, Lee M-T, Tsai M-Y, Chu C-H, Lin S-J (2014). High thermal conductive diamond/Cu–Ti composites fabricated by pressureless sintering technique. Applied Thermal Engineering.

[11] Hu H, Kong J (2013). Improved Thermal Performance of Diamond-Copper Composites with Boron Carbide Coating. Journal of Materials Engineering and Performance.

[12] Ma S (2017). Mo 2 C coating on diamond: Different effects on thermal conductivity of diamond/Al and diamond/Cu composites. Applied Surface Science.

[13] Schubert T (2008). Interfacial characterization of Cu/diamond composites prepared by powder metallurgy for heat sink applications. Scripta Materialia.

[14] Shen X-Y, He X-B, Ren S-B, Zhang H-M, Qu X-H (2012). Effect of molybdenum as interfacial element on the thermal conductivity of diamond/Cu composites. Journal of Alloys and Compounds.

[15] Zhang C, Cai Z, Wang R, Peng C, Feng Y (2017). Enhancing densification capacity and properties of Al/diamond composites by partial liquid hot pressing. Surface and Coatings Technology.

[16] Zhang Y, Zhang HL, Wu JH, Wang XT (2011). Enhanced thermal conductivity in copper matrix composites reinforced with titanium-coated diamond particles. Scripta Materialia.

[17] Jiang L (2015). Interfacial characteristics of diamond/aluminum composites with high thermal conductivity fabricated by squeeze-casting method. Materials Characterization.

[18] Zhang C (2016). Microstructure and thermal properties of Al/W-coated diamond composites prepared by powder metallurgy. Materials & Design.

[19] Li G, Xiong B (2017). Effects of graphene content on microstructures and tensile property of graphene-nanosheets/aluminum composites. Journal of Alloys and Compounds.

[20] Wu JH, Zhang HL, Zhang Y, Li JW, Wang XT (2013). The role of Ti coating in enhancing tensile strength of Al/diamond composites. Materials Science and Engineering: A.

[21] Zhang L (2018). Microtopography and mechanical properties of vacuum hot pressing Al/B_4_C composites. Ceramics International.

[22] Xu ZG, Jiang LT, Zhang Q, Qiao J, Wu GH (2017). The microstructure and influence of hot extrusion on tensile properties of (Gd + B_4_C)/Al composite. Journal of Alloys and Compounds.

[23] Chen HS (2018). Microstructure evolution and mechanical properties of B_4_C/6061Al neutron absorber composite sheets fabricated by powder metallurgy. Journal of Alloys and Compounds.

[24] Shen Q (2014). Microstructure and mechanical properties of Al-7075/B_4_C composites fabricated by plasma activated sintering. Journal of Alloys and Compounds.

[25] Rana HG, Badheka VJ, Kumar A (2016). Fabrication of Al7075/B_4_C Surface Composite by Novel Friction Stir Processing (FSP) and Investigation on Wear Properties. Procedia Technology.

[26] Sun Y (2017). Enhanced tensile strength and thermal conductivity in copper diamond composites with B_4_C coating. Scientific reports.

[27] Tan Z (2016). Tailoring interfacial bonding states of highly thermal performance diamond/Al composites: Spark plasma sintering vs. vacuum hot pressing. Composites Part A: Applied Science and Manufacturing.

[28] Wang P (2015). Enhanced thermal conductivity and flexural properties in squeeze casted diamond/aluminum composites by processing control. Materials & Design.

[29] Sun Y (2017). Reduced Graphene Oxide Reinforced 7075 Al Matrix Composites: Powder Synthesis and Mechanical Properties. Metals.

[30] Yakel H (1973). Lattice expansions of two boron carbides between 12 and 940 °C. Journal of Applied Crystallography.

[31] Hasselman DPH, Johnson LF (1987). Effective Thermal Conductivity of Composites with Interfacial Thermal Barrier Resistance. Journal of Composite Materials.

[32] Yang W (2017). Enhanced thermal conductivity in Diamond/Aluminum composites with tungsten coatings on diamond particles prepared by magnetron sputtering method. Journal of Alloys and Compounds.

